# The Myth of Colchicine in Treating Myopericarditis: Case Report and Literature Review

**DOI:** 10.7759/cureus.8933

**Published:** 2020-06-30

**Authors:** Rana Al-Zakhari, Gautham Upadhya, Sean Galligan, Fiona Shehaj

**Affiliations:** 1 Internal Medicine, Richmond University Medical Center, Staten Island, USA; 2 Cardiology, State University of New York Downstate Medical Center, Brooklyn, USA; 3 Cardiology, Richmond University Medical Center, Staten Island, USA

**Keywords:** myopericarditis, echocardiography, cardiac magnetic resonance imaging, colchicine

## Abstract

Myopericarditis is inflammation of the pericardium with concurrent myocardial involvement. The clinical presentation of myopericarditis is often with varying degrees of cardiac symptomatology. Its etiology is often idiopathic, but it may also be related to infectious and inflammatory prodrome. Symptoms are proportional to the extent and pattern of myocardial involvement. Many are diagnosed sub-clinically during the management of other systemic illnesses. Echocardiography and cardiac magnetic resonance imaging are important tools in the evaluation of myopericarditis, as the diagnosis of left ventricular dysfunction greatly affects the management, follow-up, and prognosis of these patients. The acute management of myopericarditis remains without clear direction and focuses on symptom control. The use of NSAIDs is often cautioned, as it has been described to actually accelerate the myocarditic process in animal models, possibly increasing mortality. Colchicine, a well-established anti-inflammatory agent, may have a role in the management of acute myopericarditis.

We present two cases, each involving a young male, without underlying medical conditions, who presented to the emergency room with acute onset chest pain. Both were found to have elevated cardiac biomarkers and electrocardiographic (EKG) changes, admitted as in-patients and eventually diagnosed with acute myopericarditis. They made full recoveries and were eventually discharged home. Both were started on colchicine during hospitalization, which were continued for several months upon discharge. Overall, there is limited published data regarding the medical management of myopericarditis. There need to be prospective studies and registries to further our knowledge in the management of this illness.

## Introduction

Myopericarditis is a term used for the inflammation of pericardium with concurrent involvement of the myocardium. The typical manifestations of pericarditis include positional or pleuritic chest pain or both, fatigue, decreased exercise tolerance, and palpitations. Myocarditis may be identified by imaging studies, such as cardiac MRI and other modalities that illustrate the normal ventricular wall motion [1]. Since myopericarditis is not responsible for any major myocardial dysfunction, but it might be a cause of the myocarditis-dominant syndrome, which is usually referred to as “perimyocarditis” [2].

## Case presentation

Case 1

A 23-year-old male presented to the emergency department with complaints of substernal chest pain since 10:30 in the morning while he was at work. The pain was described as substernal in location and pressure-like in nature, as if “someone sitting on his chest.” He denied any other associated symptoms. He described having an episode of loose stool the day prior, with fevers and chills since the last 2-3 days. He denied any prior medical history, surgical history, or relevant family history. Due to the persistence and severity of the pain, he called emergency medical services, to come to the ED for evaluation.

**Figure 1:**
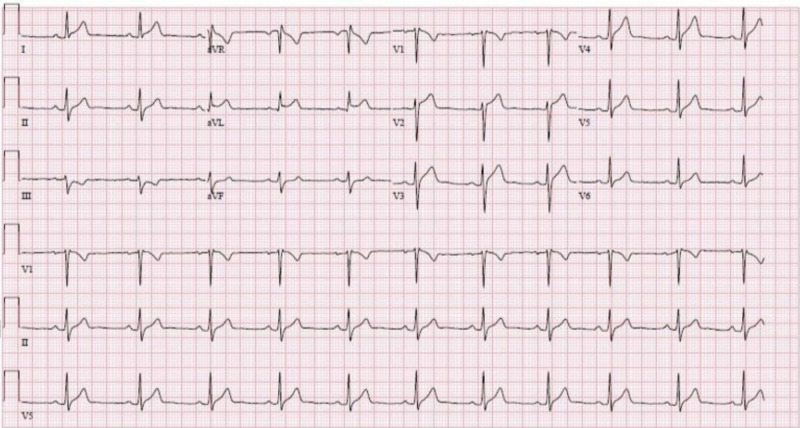
Twelve-lead surface EKG with ST-elevations in leads I and aVL (December 12, 2019)

At presentation, vitals were significant for tachycardia. Physical exam was without any discernible murmurs, rubs, or gallops. Laboratory measurements were notable for an elevated troponin I of 9.25 ng/ml (reference range: < 0.045 ng/ml). Electrocardiograph (EKG) had ST elevations in I and aVL (Figure 1). Bedside transthoracic echocardiogram (TTE) had normal systolic function without any overt wall motion abnormalities. The patient was initially loaded with acetylsalicylic acid (ASA) 325 mg, Plavix 300 mg, and Lovenox 1 mg/kg and admitted to the coronary care unit (CCU) for the management of acute coronary syndrome versus myopericarditis.

During hospitalization, troponin I peaked at 18.1 ng/ml, before down-trending to around 4.8 ng/ml. The viral panel sent was unremarkable. Inflammatory marker, C-reactive protein (CRP), was elevated to 4.56 mg/dl (reference range: < 0.29 mg/dl). Official transthoracic echocardiogram was obtained, which revealed a normal left ventricular ejection fraction of about 65%, without any focal wall motion abnormalities, valvular lesions, nor pericardial effusion. Due to lower suspicion of an acute coronary syndrome, dual antiplatelet therapy and anticoagulation were discontinued, and the patient was started on colchicine 0.6 mg twice per day (BID) for suspected inflammatory chest pain, likely secondary to post viral myopericarditis.

The patient’s symptoms improved, and he was discharged after four hospital days on colchicine BID, to be continued for 2-3 months, with outpatient cardiology follow-up. Cardiac MRI was obtained, which revealed normal bi-ventricular wall motion and normal ejection fraction. There was no evidence of scarring or residual myocarditis. The patient remained chest-pain free at all follow-ups. Colchicine was discontinued after completing its course, and the patient was cleared to resume normal activities and instructed to follow-up with his primary medical physician.

Case 2

A 36-year-old male presented to the emergency department for intermittent chest pain over the prior four days. The pain was substernal, pressure-like in quality with radiation to the left shoulder. The pain was not aggravated by movement, and intermittent in nature, lasting for several hours at a time. He stated to have had associated nausea and shortness of breath. He reported having had an episode of loose stool the day prior. He also reported that he had a newborn child and that his wife was also recently hospitalized for a gastrointestinal infection. He denied any medical history, surgical history, or relevant family history. Due to the worsening pain over several days, he decided to present to the ED for evaluation.

**Figure 2:**
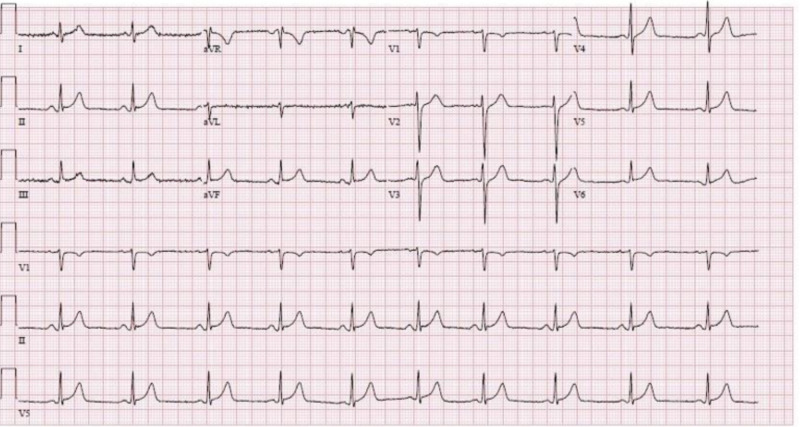
Twelve-lead EKG with 1 mm ST-elevations in leads II, III, aVF, V5, and V6 (December 24, 2019)

**Figure 3:**
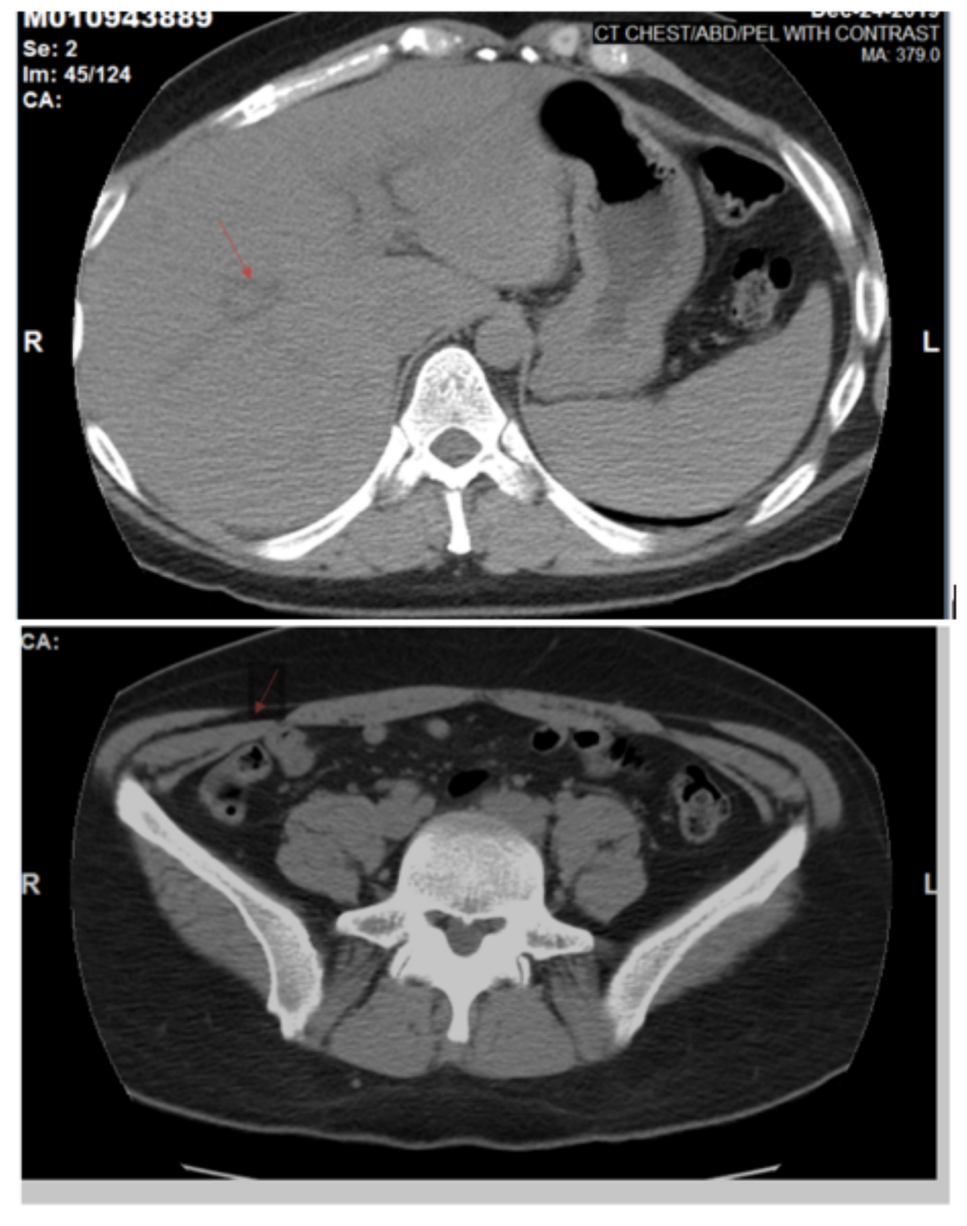
CT abdomen (top) and pelvis (bottom) revealed periportal edema with free fluid in the pelvis suggestive of an infectious and/or inflammatory process such as gastroenteritis

At the time of presentation, the patient was afebrile and hemodynamically stable and free of chest pain. Physical exam was without any acute abnormalities, no audible murmurs, rubs, or gallops. Initial laboratory tests were significant for troponin I of 2.63 ng/ml, which trended up to 8.48 ng/ml, a few hours after the initial test (reference range: < 0.045 ng/ml). EKG had concave 1 mm ST-elevations in leads II, III, and aVF and leads V5-6 (Figure 2). CT angiogram of the chest with pulmonary embolism (PE) protocol was obtained, which was negative for PE or any other acute pulmonary findings. CT abdomen and pelvis revealed periportal edema with free fluid in the pelvis suggestive of an infectious and/or inflammatory process such as gastroenteritis (Figure 3).

The patient was loaded with ASA 325 mg, Plavix 300 mg, and Lovenox 1 mg/kg and admitted to the coronary care unit for management of acute coronary syndrome versus acute post viral myopericarditis.

During hospitalization, the troponin trended downwards and leveled around 2 ng/ml. Transthoracic echocardiogram revealed an overall normal left ventricular systolic function of about 70-75%, without any focal wall motion abnormalities. Viral panels were unremarkable. The inflammatory marker, erythrocyte sedimentation rate (ESR), was elevated to 19 mm/hr (reference range: 0-15 mm/hr). Due to suspected etiology of post-viral myopericarditis, with low pretest probability for ACS, full-dose anticoagulation, aspirin and Plavix were discontinued. The patient was started on colchicine 0.6 mg BID, to be continued for 2-3 months, and discharged with outpatient cardiology follow-up. The total length of hospitalization was two days.

At one month follow-up, the patient remained chest-pain free, but with complaints of non-specific fatigue below his baseline. The patient was maintained on colchicine 0.6 mg BID, to be continued for a total of 2-3 months, with a plan for repeat transthoracic echocardiogram and laboratory tests, including iron and thyroid panels.

## Discussion

Myocarditis is an inflammatory disorder of the myocardium, which may be confirmed by clinical biopsy or more commonly by symptomatology and relevant laboratory markers [3]. When the inflammation primarily involves the pericardial sac, the syndrome is referred to as pericarditis. The inflammation of the myocardium and pericardial sac together is known as myopericarditis. However, this diagnosis may be further subdivided as myocarditis or pericarditis predominant. The majority of cases remain idiopathic, but some are related to certain infectious and inflammatory processes. Viral infections are the most common cause of myocarditis in the developed world. The most common in North America are coxsackievirus, adenoviruses, cytomegalovirus, Epstein-Barr virus, influenza, hepatitis A and C, varicella-zoster, and parvovirus B19 [2]. Metabolic, traumatic, autoimmune, and neoplastic conditions are the most causes of non-infectious myocarditis [4].

Myopericarditis is far more common in young male patients. Patients frequently report recent gastrointestinal or respiratory symptoms or both, often with associated malaise, fever, and myalgias. The extent and pattern of myocardial involvement are key factors in the clinical investigation and presentation of the illness, as many cases are diagnosed based upon subclinical studies in addition to systemic symptoms. Typical symptoms include pleuritic and/or positional chest pain, palpitations, and fatigue. Generally, one cannot differentiate myopericarditis from pericarditis based only on clinical symptomatology. Moreover, myopericarditis related chest pain is difficult to distinguish from ischemic chest pain, especially in the setting of elevated cardiac biomarkers and coronary artery disease risk factors [5].

The physical examination is often unremarkable in patients with myopericarditis. However, the presence of positive physical findings may help delineate the severity and extent of organ involvement. Such findings include fever, pedal edema, rales, gallops, and pericardial friction rubs. Workup of suspected myopericarditis includes EKG, laboratory tests including serum troponin, ESR and CRP, echocardiogram. In some cases, cardiac magnetic resonance imaging (CMR), coronary angiography, and myocardial biopsy may be indicated [5].

There are typical EKG findings that may be associated with the diagnosis of pericarditis/myopericarditis. These findings include diffuse ST-segment elevations, PR-segment depressions non-specific T-wave inversions. EKG changes more specific to that of myocarditis include localized ST elevations in the inferolateral and anterolateral leads with concordant reciprocal depressions, new Q waves, convex ST-segment morphology, and prolonged QT interval. Irregular heartbeats such as atrial premature complexes (APCs), ventricular ectopy and/or unstable ventricular tachycardias are more closely associated with myopericarditis [5].

Myopericarditis causes an elevation in creatine kinase-MB (CK-MB) fraction and/or troponin I /T (cardiac biomarkers). The degree of myocardial injury is directly proportional to the magnitude of troponin elevation. For reference, the positive predictive value and reported sensitivity of troponin in the diagnosis of myopericarditis are 90% and 34%, respectively [1]. The pattern of troponin elevation usually begins with a mild increase followed by either a rapid decrease or a further elevation similar to that of an acute coronary syndrome. In less than five percent of cases, there may be a persistent elevation of troponin levels that may last for up to several weeks. This phenomenon may represent sustained myocardial injury and deterioration [5].

Echocardiography is critical in the identification of ventricular dysfunction, myocarditic thrombi, valvular incompetence. Pericardial involvement can range from almost none, up to cardiac tamponade in some clinical situations. Myopericarditis, by definition, does not involve significant myocardial dysfunction. However, the presence of severe and/or prolonged ventricular dysfunction represents the severe myocarditis-dominant syndrome, perimyocarditis [6,7].

Pericarditis and myocarditis can be diagnosed accurately with the aid of cardiovascular magnetic resonance imaging (CMR). MRI imaging techniques used in the identification of myocardial inflammation utilizes T2 weighted studies for the assessment of myocardial edema and hyperemia. Delayed gadolinium enhancement can be evaluated by T1 weighted imaging, which consists of pre and post-contrast studies. Two out of three MRI imaging techniques that are positive for myocardial inflammation is confirmatory of its diagnosis. MRI imaging also has the ability to differentiate acute coronary syndrome from other inflammatory conditions [2].

Despite endomyocardial biopsy being considered the gold standard in the diagnosis of myocarditis, there are only a few indications for performing it. Patients with subacute or acute heart failure refractory to medical therapy; heart failure associated with rash, fever or peripheral eosinophilia; hemodynamically significant arrhythmias; suspected giant cell myocarditis; a history of collagen vascular diseases are all recommended to undergo myocardial biopsy [2].

In many patients, it is difficult to differentiate myopericarditis from acute coronary syndrome. Some patients should be considered for invasive or noninvasive ischemic testing. Patients identified as having a high pretest probability for obstructive coronary artery disease would benefit the most from an early invasive strategy, such as coronary angiography. No invasive workup was done for our patients as both have a low pretest probability, and both have achieved full recovery after a few months of treatment with colchicine [8].

Viral and other infectious serological panels are usually not recommended as part of the initial workup of myopericarditis or pericarditis. The vast majority of cases are among immunocompetent patients from the developed countries, of which the etiologies are often idiopathic [9]. Therefore, extensive laboratory testing is not only cost-ineffective but also adds little to no value to patient care.

General guidelines should be followed for the evaluation and diagnosis pericarditis even in higher-risk patients who may present with fever, subacute onset, immunosuppression, trauma, those on oral anticoagulant therapy, or with large pericardial effusions [10]. More exhaustive workup inclusive of the serologic studies may be pursued when routine studies fail to provide a diagnosis to provide treatment. 

There is limited data available regarding the treatment of myopericarditis. When myopericarditis presents with preserved ventricular function, without the presence of significant ventricular arrhythmias, it is managed in a similar fashion to that of acute pericarditis, which primarily entails the use of high dose non-steroidal anti-inflammatory drugs (NSAIDS) [1,2]. However, in several animal models, the use of NSAIDs in the setting of myocardial inflammation, had a detrimental effect, increasing mortality rates and possibly exacerbating the myocarditic process [11-13].

One possible treatment modality in the management of myopericarditis is to provide the lowest possible dose of NSAIDs in order to control symptoms. In the setting of global and/or regional left ventricular dysfunction, the use of beta-blockers and ACE-inhibitors is advocated [14]. At this time, there is no conclusive data that the use of colchicine provides any significant benefit in the management of myopericarditis. However, it is often used on a case-by-case basis in clinical practice.

Among myopericarditis patients who develop significant left ventricular dysfunction, guideline-directed management and expert cardiology consultation are recommended. In such cases, non-specific measures, such as the restriction of intense physical activity (i.e., competitive sports) for at least three months is often recommended, with serial medical assessments for up to 12 months [15].

## Conclusions

Our two cases involved two otherwise healthy patients, who developed acute myopericarditis, likely secondary to unspecified viral illnesses. Both patients required short inpatient hospitalizations and made full uneventful recoveries. Both patients had viral panels sent, which were non-diagnostic, and started on colchicine, which may or may not have aided their recovery. Myopericarditis remains a commonly diagnosed syndrome that often remains without any clear diagnostic algorithm. With this paper, we hope to review different approaches to the diagnosis and management of myopericarditis, as well as create a discussion regarding possible alternative treatment modalities.
